# Unravelling proteinopathies: an interview with David Rubinsztein

**DOI:** 10.1242/dmm.021360

**Published:** 2015-07-01

## Abstract

David Rubinsztein is currently professor of Molecular Neurogenetics at the University of Cambridge, UK and Wellcome Trust Principal Research Fellow. He is based in the Cambridge Institute for Medical Research, where he is currently the Deputy Director. He started his studies in medicine in Cape Town, South Africa, and was initially interested in both clinical and research work. During his PhD, he discovered himself truly passionate about cell biology and genetics of disease and moved to Cambridge, where he specialised in Genetic Pathology, after which he received a 6-year Glaxo-Wellcome Fellowship to investigate mechanisms of protein misfolding and aggregation in Huntington's disease and other proteinopathies. Since then, he has been committed to lab research and has been a leading scientist in elucidating the roles of autophagy in neurodegeneration. In his lab, he combines cell biology tools with animal studies to elucidate the potential of autophagy manipulation as a strategy to eliminate toxic misfolded and aggregated proteins and treat neurodegenerative diseases. He has been recently appointed as academic lead PI for the Alzheimer's Research UK Drug Discovery Institute in Cambridge, whose goal is to develop disease-modifying treatments for neurodegenerative diseases. In this interview, David tells us how he developed his career as an independent scientist, sharing his experience and views about the scientific progress in our understanding of neurodegenerative diseases and in developing potential therapeutics.

David Rubinsztein was born in South Africa. He obtained his Bachelor of Medicine and Surgery in 1986 at the University of Cape Town and at the same institute he completed his PhD in 1993. He then moved to Cambridge where he completed his specialist training in Genetic Pathology in 1997. After this he was awarded a 6-year Glaxo-Wellcome Fellowship, which allowed him to very quickly set up his independent research, trying to understand the pathophysiology of Huntington's disease and other neurodegenerative diseases related to protein misfolding and aggregation (the so-called proteinopathies). He then received a Wellcome Trust Senior Fellowship in Clinical Science followed by a Wellcome Principal Fellowship, providing the opportunity to further develop his own group. His lab revealed that autophagy can modulate neurodegeneration in conditions like Huntington's disease and discovered autophagy manipulation as a potential therapeutic strategy for various proteinopathies. As academic lead PI of the Alzheimer's Research UK Drug Discovery Institute in Cambridge, he hopes to accelerate progress towards new treatments for neurodegenerative diseases.

**Figure:**
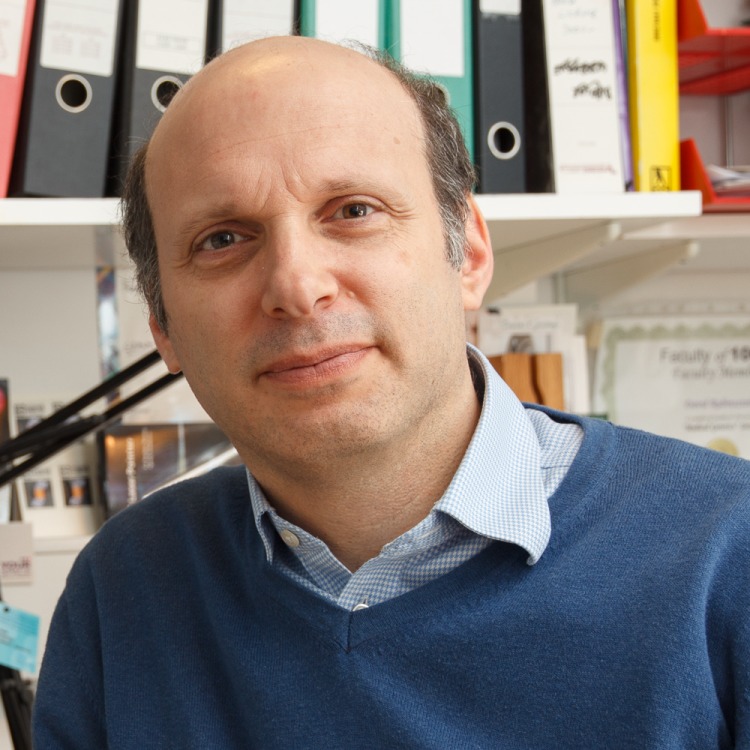

“I realised that one way to have sufficient time to devote to research was to obtain independent fellowship funding”

**You initially received a medical training but then decided to fully commit to research work. Why was that?**

When I was young, I thought I wanted to be a traditional academic clinician – somebody who did quite a lot of clinical work and a lot of research. I thought there was a way of doing both. After I did my medical training, I first did a BSc Honours – so I spent a year doing a smaller project and learning some more science – and then I did my PhD. About halfway through my PhD, I realised that if I wanted to do the type of work I was interested in, predominantly lab research investigating cell biology and cellular mechanisms, then it would be very difficult to do that part time, as you would compete with people who do it full time. At that point I thought I had to strategise my career to fulfil my objective. Then I came to Cambridge and I was very lucky as I was the first trainee in Cambridge, in the country actually, doing pathology with genetics. From that point on, I realised that one way to have sufficient time to devote to research was to obtain independent fellowship funding.

**What were your initial scientific interests and how did you establish your independent research?**

I worked initially on the LDL [low density lipoprotein] receptor and the biological consequences of its mutations. That was what my PhD was mainly about. When I came to Cambridge, I was recruited by Malcolm Ferguson-Smith in a genetic training position, which was actually a clinical position but I had a lot of time to dedicate to research. Those were the days when you didn't have to ‘tick so many boxes’, so I could do research and get accredited in my specialty. Before I finished that, I got a Glaxo-Wellcome Fellowship – it was the first round of them – and that gave me a start as an independent researcher. After 4 years, I was awarded a Wellcome Trust Senior Fellowship in Clinical Science (which was renewed after 5 years) and then I received a Wellcome Trust Principal Fellowship. These fellowships have given me the freedom to spend most of my time doing research – which has been wonderful! I'm really grateful to the people who funded my research. It would have been extremely difficult otherwise.

**Why did you move to Cambridge?**

At the time, I didn't know what I know now about Cambridge. But I'm so happy that I moved here. Ferguson-Smith – who became my boss – was interested in fostering people who were involved in academic medicine, and that was important to me. He had a lot of energy and I thought there was an opportunity for a job where I could get some more clinical training and still pursue science because the two often are almost mutually exclusive or very difficult to do together. Now of course, in retrospect, I appreciate so much that Cambridge has got a very rich scientific environment and there is a lot happening. Although it is a small town still, there's a tremendous amount of great science going on. I came from Cape Town where, if you want to do something new, you could be the only person in the country doing that, but in Cambridge, if you have an experimental problem or an interesting question to address, you can discuss it with other experts and there's really sufficient critical mass and scientific excellence to provide the backdrop for trying to do interesting work.

**Are collaborations important for your research?**

They are extremely important. When I was younger I collaborated a lot with Bill Amos, and together we did some studies on population genetics. I've collaborated extensively with the *Drosophila* geneticist Cahir O'Kane. We do quite a lot of zebrafish work in my lab, and in fact it started with the collaboration we had with DanioLabs, a company that was set up in Cambridge. We also collaborate very closely with Roger Barker, an excellent neurologist who does a lot of clinical work, and with Andres Floto, who works on tuberculosis; together we are studying autophagy in tuberculosis. I have also had excellent collaborations with Steve Brown, an outstanding mouse geneticist, and with pharma and biotech. The most recent of these, with Siena Biotech, led to the discovery of a novel druggable target that reduces the aggregation rate of mutant huntingtin in cells and *in vivo* – the paper has just come out in *Nature Chemical Biology*. These collaborators have all helped enrich the work from my lab in various ways, both providing intellectual support and novel areas of expertise.

**Why did you decide to work specifically on neurodegenerative diseases?**

I think the reasons have evolved in a way. When I came to Cambridge, very soon after I had been in the lab, the Huntington's disease gene was identified by a large international consortium led by James Gusella at Harvard. I had always been interested in this disease, even before I knew much about diseases caused by triplet repeats, because the underlying genetic mechanism was very interesting – at the time, it was quite unexpected genetics. I had been in the lab for a month or two and when the gene was cloned, the person who was the head of the diagnostic lab put all the DNA samples he had access to from the local clinic on my desk. He said: “here are the DNAs, see what you can do”. I did some experiments and I found that the initial diagnostic assay that was described in the original paper on the Huntington's gene wasn't absolutely accurate. Huntington's disease is caused by the expansion of polymorphic triplet repeats. The initial paper assumed that only one set of repeats in the gene was polymorphic in triplet repeat number but I found that an adjacent set of triplet repeats included in the original PCR assay was also polymorphic. This had a potential confounding effect, albeit minor, on the assay that was proposed for clinical diagnosis. That was not a great discovery but I got hooked a little bit on this field, although in a sense that was just chance.

Now, over the 20 or more years I've been in Cambridge, I think that studying neurodegenerative diseases is the right thing for me. I think these are critical diseases for our society. Many excellent labs have identified genes and variants that impact on the diseases and some pathogenic pathways, so I think there are possibilities for developing therapeutic strategies for some of these diseases. Some of them are quite easy to model; for example, you can model Huntington's disease with a reasonable fidelity compared to many other diseases by using cell models and a range of animal systems. Now, I am very committed to it and I hope that some of the approaches we are taking might have value across at least a few neurodegenerative diseases. So what started as a chance has evolved into something where we have a very big commitment.

**Could you tell us how the protein-misfolding disease mechanism was discovered for Huntington's disease?**

There is a paper buried in the literature from Jim Lowe and colleagues in 1995 in *Neuropathology and Applied Neurobiology* suggesting that there were ubiquitinated aggregates in Huntington's disease brains. But the people who made the big impact with this were Steve Davies, Gill Bates, Marion DiFiglia and Neil Aronin, who, in 1997, described ubiquitinated aggregates in Huntington's patients and Huntington's mice. At the same time, Erich Wanker described the phenomenon in cell-based systems. Of course that was very important because it was consistent within the existing literature of protein aggregation in a whole group of other diseases, like Alzheimer's disease and prion diseases. That really did open the door to thinking about therapeutic strategies for the disease and also disease mechanisms, which we still don't understand that much about.

**What was the most exciting discovery in your career?**

I think when we discovered that the aggregate-prone proteins that cause neurodegenerative diseases are autophagy substrates, and that we could enhance their removal by upregulating autophagy. That discovery sort of opened the door to a whole dimension that we hadn't anticipated. We were not interested in autophagy 14-15 years ago. That was a period where my lab worked mainly on neurodegeneration and did very little on autophagy. Now the lab is really focused on autophagy, which ended up being a core expertise of the lab. And I think that's the most exciting work that we have done.

**So modulating autophagy could be a potential therapeutic strategy for proteinopathies. How far are we from the clinical translation of this strategy?**

It is more a question of what the timelines of the rate-limiting steps are. At the moment we have a drug in a safety trial for Huntington's disease. But we think that there are quite a few drugs (many of which are FDA approved) that potentially would be able to regulate autophagy in humans. At certain concentrations, we found that they can do so in cell-based systems, in *Drosophila* and in mice. At the moment, we are making quite a big effort to try to understand if the concentrations of these drugs that cause upregulation of autophagy either in the cells or the animal models are achievable in man. That involves quite careful pharmacokinetic studies linked with careful assays of autophagy and aggregate substrate accumulation in *in vivo* models. This is harder than I hoped it would be, but I think it's the problem everyone has and can be tractable. For example, one problem is that we are using mainly mice for such studies: often in mice the half-life of a drug is about 30 minutes, while the same drug has a half-life of 10 hours in people. We need to find ways of getting around that, but we can use osmotic pumps to deal with that. Those experiments will take a year or two, but we can get the answers to our questions. What is going to be hard is doing clinical studies. A preclinical study in mice can take a year. A clinical study in humans takes much longer and you need a much larger sample size because of the disease heterogeneity. Human studies are thus the rate-limiting step. I hope we will be able to build a portfolio of drugs that we have validated carefully as having worked in mice, so that we can start testing these in humans. We will need time to find financial support, we need time to go through regulatory procedures in humans but actually the major delay will be the difficulty in doing the trial properly. I am worried that many of the trials in humans for these diseases are done too late in the disease course and sometimes with insufficient preclinical data to know whether the doses are going to be sufficient.

**What would you like to achieve in the next 10 years?**

Starting with the translational work, I think that we should look at molecules and mechanisms that we, and others, have already described, as there might be potentially useful targets for drugs, and see if they would meet the criteria for consideration in human studies. There are possibilities out there, and we sometimes don't need to make a new discovery but we just need to reconsider already available compounds. That's comparatively straightforward at least intellectually; practically, there's some work involved. Of course, if these approved drugs do not end up being suitable, then we need to find new drugs that can attack these mechanisms.

Then, I think we need to understand which diseases are most suitable for autophagy manipulation and this requires further experiments and understanding of disease mechanisms. Modelling complex diseases such as sporadic Parkinson's and sporadic Alzheimer's is challenging. I think we need to get an idea of what is going on there. I am very interested in understanding if and how autophagy might be compromised in these types of complex diseases and with this knowledge we might be able to understand if autophagy modulation is a suitable strategy for these diseases.

“…we sometimes don't need to make a new discovery but we just need to reconsider already available compounds”

We are making a big effort in my lab to try to understand the basic biology of autophagy and there are two important aspects that we are trying to address. One is how autophagosomes form, the nuts and bolts in the machinery, and there is still quite a lot of work to sort out there. This will provide a backdrop for understanding disease pathogenesis. For example, previously we described the trafficking routes of vesicles containing different autophagy proteins that ultimately fuse to give rise to autophagosome precursors and used this basic information to understand how these processes were disturbed by a mutation that causes Parkinson's disease and by a gene in a GWAS locus for Alzheimer's disease. Thus, the basic biology can really provide a backdrop for disease-associated studies. The second aspect we are also very interested in is autophagy signalling. For instance, we have recently published a paper in *Molecular Cell* about PI(5)P [phosphatidylinositol 5-phosphate] regulating autophagy and we identified some enzymes that might be potential targets to manipulate for therapeutic purposes.

What I would like to do over the next period is to pursue both the basic research work, which I believe really provides the foundation for developing therapeutics and understanding pathogenesis, alongside the clinical angle, which I think is very exciting and for which there are a lot of possibilities, not only for neurodegenerative diseases: I mentioned the work that I am doing with my colleague Andres Floto in the field of multi-drug resistant forms of tuberculosis for which there might be a promise for autophagy upregulation, as well – we recently published a paper on this aspect in *EMBO Molecular Mechanism*. A success with any disease would be wonderful.

**Alzheimer's Research UK has very recently (February 2015) launched its Drug Discovery Alliance, allocating £30m to sustain three Drug Discovery Institutes in Oxford, Cambridge and London. The goal is to develop disease-modifying treatment for dementia. As lead academic of the Drug Discovery Institute in Cambridge – our sincere congratulations! – do you think this is a feasible target? What are the ingredients that could make this real?**

These institutes aim to find therapies for neurodegenerative diseases, including but not restricted to Alzheimer's disease. The vision of Alzheimer's Research UK is bold and innovative, in trying to embed this drug discovery effort within academic centres. My view is that this is a novel approach that has a real chance of making headway by combining the strengths of academic institutions and the expertise of pharma and biotech in drug development. This potential will be further enhanced by the alliances and cooperativities between the Cambridge, Oxford and UCL Institutes, which have complementary strengths. I should stress that we are recruiting a Chief Scientific Officer who will run the Cambridge institute *de facto*, so my role will be as an advisor, supporter and collaborator. I am really excited about the potential for developing the translational potential of more basic discoveries more rapidly and effectively in this type of model.

**In your long career is there a particular person who has influenced you?**

I have learnt many things from many people. I started off in Cape Town and the head of the department, Wieland Gevers, was very influential in that he provided an example of what an intellectual academic would be. My supervisors, Denys van der Westhuyzen and Gerry Coetzee, taught me how to do science, they loved science. I think that is the most important thing they taught me – they really had a passion for science in all its senses, they had a very good and honest attitude about doing science. Then I came to Cambridge and Ferguson-Smith was very influential. He is a man with tremendous energy. He was succeeded by Martin Bobrow and Martin was very supportive, as well. Martin also made me realise the importance of common sense – he has this enviable knack of identifying the key issues and not getting lost in the peripheral distractions. Then many of my colleagues in Cambridge and further afield have been inspiring in diverse ways, as are my students and postdocs!

“…there are very few careers where you are pretty much the master of your own destiny even as a student or a postdoc. You can really discover things and, occasionally, there is the potential to make a difference”

**Do you have any advice for young scientists? What do you recommend to your students and young collaborators for example?**

I'll tell you what I told them yesterday. You do science with your head and one way to fuel scientific creativity is to be really immersed in the domain you're working in, to know the literature well and read the literature critically. People work hard but what matters is that you think hard as well. If you're interested in making a lot of money, this is not the career to do. But looking at the positive things, there are very few careers where you are pretty much the master of your own destiny even as a student or a postdoc. You can really discover things and, occasionally, there is the potential to make a difference. We all strive for that, but it's unpredictable. Some of the things that look most plausible on paper end up being intractable at the last step. The advice is to be immersed in it and have fun. It's hard work but fun. And in this regard, this can be the best job in the world.

**You are really passionate about science, but if not science what career would you have pursued?**

If I think about what would give me a kick if it weren't science, medicine or something academic, I would say being an orchestral conductor. Although, being a lab head is pretty much the same: you conduct, you don't play instruments anymore.

**What do you like doing outside the lab?**

I play the cello and the piano, and I like listening to music. I have kids, so I enjoy spending my time with them and my wife.

